# A Protein-Based Approach for Greek Yogurt Authentication via an HRMS Technique (MALDI-TOF MS) and Milk Powder Detection as a Fraudulent Addition

**DOI:** 10.3390/foods14040693

**Published:** 2025-02-18

**Authors:** Evangelia Krystalli, Nikolaos Thomaidis, Anastasia S. Kritikou, Christos Kokkinos

**Affiliations:** 1 Laboratory of Analytical Chemistry, Department of Chemistry, National and Kapodistrian University of Athens, Panepistimiopolis Zografou, 15771 Athens, Greece; evakryst@chem.uoa.gr (E.K.); ankritik@chem.uoa.gr (A.S.K.); christok@chem.uoa.gr (C.K.); 2 Hellenic Research & Innovation Center (HRIC), YIOTIS S.A., 12131 Athens, Greece

**Keywords:** MALDI-TOF MS, Greek yogurt, authenticity, protein profile, adulteration

## Abstract

The popularity of Greek-style yogurt (made from cow, ewe, and goat milk) has grown significantly in recent years thanks to its high protein content, nutritional value, and unique creamy texture, making it vulnerable to illegal practices, such as adulteration. In the present work, a fast and reliable matrix-assisted laser desorption/ionization time-of-flight mass spectrometry (MALDI-TOF MS)-based methodology was developed for the detection of yogurt adulteration with cow milk powder, exploiting the intact protein profile. An integrated protein-based workflow was established for the detection of as little as 1% cow milk powder addition into cow and goat milk yogurt. Simultaneously, markers for yogurt classification based on their animal origin (cow, ewe, or goat), type (traditional or strained), and thermal treatment of milk were revealed for the first time. Statistical analysis using chemometric tools, such as unsupervised principal component analysis (PCA) and supervised partial least squares discriminant analysis (PLS-DA) recognition techniques, were implemented for the discrimination/classification of the yogurt samples.

## 1. Introduction

Dairy products are widely consumed throughout the human lifetime since their high nutritional value makes them a main source of important nutrients in the human diet. Fermented milk products constitute a fast re-growing market because the consumer has recognized their health benefits and special sensory characteristics [1], with yogurt being one of the most consumed. As described in *Codex Alimentarius*, yogurt is a fermented milk product elaborated with two specific starter cultures, *Lactobacillus delbrueckii* subsp. *bulgaricus* and *Streptococcus thermophilus*, that “shall be viable, active, and abundant (at least 10^7^ CFU/g) in the product till the date of minimum durability”. Also, it must contain at least 2.7% milk protein content and no more than 15% milk fat. Other non-dairy ingredients, such as gelatin or starch, and other milk products should not be added to its production [2,3]. Additionally, along with *Codex Alimentarius*, each country sets specific restrictions for manufacturing different types of yogurts. According to the *Greek Code of Foods and Beverages*, as described in Article 82 [4], yogurt’s minimum protein content must be 3.2% for cow and goat milk yogurt and 5.5% for ewe milk yogurt. Moreover, it is stated that during the production process, except for milk, the only added ingredients can be milk cream for lipid regulation and milk proteins in a quantity that does not affect the initial milk solids. Furthermore, the added substances should originate from the same animal species of the milk being used. Thus, the addition of milk powder is strictly prohibited. Finally, in the *Greek Code*, two yogurt types are specifically described: strained and traditional yogurt. After coagulation, the strained yogurt is drained by removing part of the whey, whereas, the traditional yogurt, also known as set yogurt [3,5], is traditionally fermented in its container, without further stirring or water removal, in order to form a membrane on its surface in the end. The minimum protein content is 5.6% for cow or goat and 8% for ewe milk strained yogurts. For traditional yogurt production, the milk should be raw or pasteurized without any modification of its natural composition, apart from lipid content regulation.

Worldwide, even though Greek yogurt is not yet registered as a PDO (Protected Designation of Origin) or PGI (Protected Geographical Indication) product, yogurts that are marketed as Greek-style are considered the most popular ones, thanks to their high protein content and unique creamy texture [5,6]. Therefore, considering that Greek yogurt is highly ranked in the global market, its wide consumption and preference make it vulnerable to adulteration. There are mainly two reasons that usually lead to fraudulent practices in the yogurt industry. First is financial gain and the second, and most important, is the achievement of proper consistency. In this scope, a common practice to achieve the proper texture is the addition of cow milk powder in all yogurt types to reach high protein content and improve the mouthfeel of the final product [7,8]. Moreover, specifically for goat and ewe milk yogurt production, the limited availability of ewe and goat milk as raw materials, combined with the lower price of cow milk, leads producers and dairy industries to adulteration practices for profit purposes. Therefore, in this case, fraud control is vital, especially during low lactation periods when the addition of cow’s milk or other additives, such as cow milk proteins or cow milk powder, is more common to achieve the necessary protein content and smooth texture. However, yogurt, particularly Greek yogurt, despite its growing popularity and high consumption, has not been thoroughly studied. To date, many studies have focused primarily on milk fraud and the authentication of PDO cheeses, such as water buffalo mozzarella, ricotta, and Feta cheese, through proteomics [9,10,11,12,13,14,15].

Recently, many methods have been developed for milk species identification in dairy products, mostly for milk and cheese. Most of them are based on milk protein analysis. It is worth noting that there is a reference method in the European Union Regulation (EU) 2018/150 involving the isoelectric focusing of γ-caseins after plasminolysis for the detection of cow’s milk in cheeses from ewe’s and goat’s milk [16]. The main drawbacks of this official method are that it is laborious, time-consuming, and lacks clear result interpretations [8]. However, there is no reference for its application in yogurt. Instead, cow milk detection in goat yogurt by electrophoresis of para-κ-casein has been proposed [17]. Other techniques that have been regularly reported for dairy product authenticity studies and are based on milk protein analysis involve reversed-phase liquid chromatography (RP-HPLC) coupled with mass spectrometry or other detectors; immunochromatographic test kits; immunoenzymatic methods, such as sandwich and indirect ELISA; DNA-based analytical methods, such as real-time PCR (Polymerase Chain Reaction); high-resolution mass spectrometry, such as matrix-assisted laser desorption/ionization time-of-flight mass spectrometry (MALDI-TOF-MS); and finally spectrometric techniques, such as near infrared (NIR) [8,9,10,11,18,19,20,21,22,23,24,25,26,27,28,29,30]. To our knowledge, specifically for yogurt, only a limited number of studies have been reported. These studies focused only on cow milk detection in ewe milk yogurt [11] and in goat milk yogurt [19,24].

Regarding the fraudulent addition of milk powder in dairy products, this adulteration technique is currently being detected through the detection of Maillard reaction products, such as hydroxymethylfurfural (HMF), fluorescent measurement at different wavelengths, the soluble tryptophan (FAST) index, enzyme activity loss, or sugars detection like lactulose [31]. However, these techniques are time-consuming and present several limitations to be applicable in the food industry. Milk powder can also be detected through the detection of the lactosylation of milk proteins directly by protein mass spectrometry, most commonly by LC-ESI-MS and MALDI-TOF-MS. However, to date, these studies have only been applied to the detection of milk powder in fresh milk, not in yogurt [32,33].

The aim of the present study was to investigate yogurt authenticity by developing an integrated MALDI-TOF MS-based workflow, exploiting the advantages of this technique in terms of specificity, sensitivity, and short analysis and hands-on time. Specifically, based on protein profiles and the exploitation of chemometric tools, the discrimination of samples by milk animal origin, as well as by technological process (traditional and strained), was achieved. Furthermore, the detection of yogurt adulteration with cow milk powder was also investigated to achieve levels as low as 1%. Similar methods have been developed only for raw milk by utilizing peptide and protein profiles [8,14,33], while their application to yogurts remains totally unexplored.

## 2. Materials and Methods

### 2.1. Samples

In order to implement possible modifications due to different animal breeds and processing techniques in the study, yogurts were obtained from 22 different producers, while milk powders were obtained from 4 different manufacturers in different periods of time. In total, 49 distinct authentic yogurt samples (8 cow milk traditional yogurts, 7 cow milk strained yogurts, 23 goat milk traditional yogurts, and 11 ewe milk traditional yogurts) were analyzed in this study. Furthermore, for the adulteration study with milk powder, 19 distinct cow milk powders were analyzed. The term “authentic” means that the samples were of known origin from various Greek producers and not adulterated. Moreover, commercially available yogurt samples were also analyzed to evaluate the applicability of the method in unknown samples. All samples were collected between November 2021 and July 2022 and analyzed fresh at that time. Yogurt samples adulterated with cow milk powder were prepared in-house in different adulteration levels (1, 5, 10 and 50% *w*/*w*), by mixing milk powder with yogurt. This approach was used due to a lack of necessary equipment to produce adulterated yogurt following the whole production process, including fermentation. Consequently, any potential metabolization of milk powder proteins during fermentation was not considered in our study. Finally, procedural blanks were also prepared and analyzed to verify the absence of any interferences.

### 2.2. Chemicals and Reagents

Sinapinic acid (SA) and Protein Calibration Standard I including the following proteins: insulin ([M + H]^+^ = 5734.51), Ubiquitin I ([M + H]^+^ = 8565.76), Cytochrom C ([M + 2H]^2+^ = 6180.99 and [M + H^+^] = 12,360.97), Myoglobin ([M + 2H]^2+^ = 8476.65 and [M + H^+^] = 16,952.30), and their dimers, resulting from the ionization process, were provided from Bruker Daltonics (Bremen, Germany). Acetonitrile (ACN) LC-MS grade was obtained from Sigma-Aldrich (Stenheim, Germany), ethanol LC-MS grade from Fluka (Buchs, Switzerland), and trifluoroacetic acid (TFA) LC-MS grade from Merck KGaA (Darmstadt, Germany). Distilled water was provided by a Milli-Q purification system (Millipore Direct-Q UV, Bedford, MA, USA).

Protein Calibration Standard I was prepared according to the product information guidelines, by dissolving 1 vial of calibration standard in 125 μL of 1:1 water and acetonitrile in 0.1% TFA and kept at 4 °C.

Two matrix solutions were prepared. A saturated SA solution in ethanol (MS1) and a saturated SA solution in a mixture of TFA 0.1%: acetonitrile, 70:30% *v*/*v* (MS2) and kept at room temperature.

### 2.3. Sample Preparation

Sample preparation was based on the method reported by Kritikou et al. [11] after proper optimization in terms of sample weight, homogenization, and dilution. Yogurt samples were first diluted 2.5-fold in 0.1% TFA, while milk powders were diluted 25-fold in 0.1% TFA. Then, a proper homogenization was achieved via 1 min vortex stirring and 20 min mechanical rotary shaking. Samples were then subjected to ultrasonication for 15 min at room temperature and afterwards centrifuged at 4000 rpm for 5 min. Finally, the aliquot was diluted 100-fold with the same solvent. For MALDI-TOF analysis, a 1:1 mixture with the matrix MS2 was prepared. From the final mixture, 1 μL was deposited on an MSP 96 target ground steel plate (Bruker Daltonics, Bremen, Germany) and dried at room temperature. In order to achieve more homogenous spots and higher sensitivity, the double-layer method for spotting was preferred. Thus, prior to sample spotting, 1 μL of matrix MS1 was deposited on the target plate and allowed to dry. Finally, to check for repeatability, sample extracts were analyzed in triplicate.

### 2.4. MALDI-TOF MS Analysis

Analysis was performed on a microflex MALDI-TOF Mass Spectrometer (Bruker Daltonik GmbH, Bremen, Germany) equipped with a nitrogen UV laser (337 nm), at 60 Hz and operated in linear detection mode with positive ion acquisition between 5 and 30 kDa *m*/*z* range. Mass spectra were automatically collected, summing 1200 shots for each sample. Random walk mode (partial spot) was used, with 50 shots collected at each raster position. The laser power was adjusted to 67% (relative power) in order to avoid detector saturation. Each sample was analyzed in triplicate. External mass calibration was performed with the Protein Calibration Standard I (Bruker Daltonics, Billerica, MA, USA).

### 2.5. Data Treatment and Statistics

MS data collected from the samples were internally calibrated using the FlexAnalysis 3.4 Bruker Software. For the statistical analysis, the raw data were extracted as mzXML files and imported to the R environment (version 4.4.2). Data pre-processing and the peak picking process were carried out following the framework developed for MALDI data processing, as previously reported in detail in the work of Kritikou et al. [11]. Briefly, for data pre-processing, a smoothing process was conducted using a Savitzky–Golay Filter with a half-width size of 15 points and order of 3, while a baseline correction was performed in raw data using Sensitive Nonlinear Iterative Peak (SNIP). For the peak picking, two methods from the MALDIquant R package were used, the Median Absolute Deviation “MAD” method and the “detectPeaks” method. Spectra visualization was achieved by the mMass 5.5.0 Software [34,35]. The pretreated data were then subjected to statistical analysis. As an initial step, a principal component analysis (PCA) was performed. The unsupervised model was used as a descriptive approach to locate any existing clustering of each dataset and facilitate data interpretation. Then, a supervised classification model, partial least square-discriminant analysis (PLS-DA), was used for the prediction and classification of unknown samples. The significant markers that were attributed to each category were identified through the calculation of the Variable Importance in Projection (VIP) score. The VIP of a variable is calculated as a weighted sum of the squared correlations between the PLS-DA components and the original variable. VIP scores larger than 1 indicate the most contributing variables [36,37].

## 3. Results and Discussion

**Figure 1:**
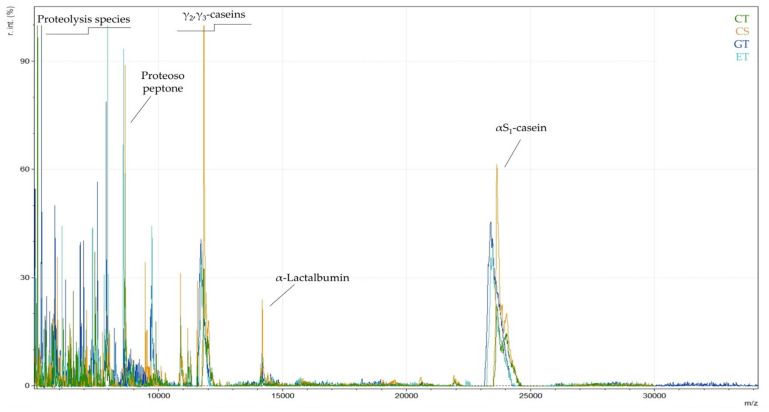
MALDI MS spectra of yogurt samples: cow milk yogurt (CT: traditional cow milk yogurt, CS: strained cow milk yogurt), goat milk yogurt (GT), and ewe milk yogurt (ET).

As a first approach, characteristic mass spectra of each yogurt type (cow milk traditional (CT), strained yogurt (CS), goat milk yogurt (GT), and ewe milk yogurt (ET)) were visually compared in order to investigate the potential workflow, identify known milk proteins, and to locate possible differentiations. Yogurt milk proteins are composed of 80% casein fractions and 20% whey proteins [38]. In our study, it was observed that yogurt samples had a high abundance of characteristic caseins, while the common whey milk proteins, α-lactalbumin and β-lactoglobulin, were absent or in low concentrations (Figure 1). The most important detected caseins were as1-casein at 23.6 kDa for cow milk yogurt and at 23.4 kDa for goat and ewe milk yogurts, γ2-casein at ~11.8 kDa for cow milk yogurt and ~11.7 kDa for goat and ewe milk yogurts. Additionally, as depicted in Figure 1, the proteose peptone at ~8.5 kDa had intense peaks. Moreover, lower-molecular-weight peaks in the region of 5 to 7 kDa were abundant in yogurt samples. These peaks have not been assigned to specific proteins and can be attributed to proteolysis during yogurt production. Proteolysis occurs either by the starter cultures or by indigenous milk proteases, such as plasmin [39]. Plasmin mainly hydrolyses β-casein to form γ-casein and proteose peptones, as well as it hydrolyses as1-casein [39,40]. The absence of whey proteins in Greek yogurts has also been reported in the work of Ruprichová (2012), where protein profiles (αs-casein, β-casein, κ-casein, α-lactalbumin and β-lactoglobulin) of different yogurt samples were analyzed by HPLC-PDA [41]. Indeed, whey proteins can be significantly reduced or absent in yogurt due to the fermentation process [42] or heat treatment [38]. First, thermal treatment before fermentation can cause extensive denaturation of whey proteins, which will interact with caseins to form micelles, preventing their free presence in yogurt. This treatment will also improve the texture of set yogurt, as it will reduce syneresis and increase elasticity [43]. Moreover, as reported by Tzvetkova (2007), the degradation of whey proteins may be a result of the fermentation process, as lactic acid bacteria can reduce α-lactalbumin concentration by up to 50% and β-lactoglobulin by 20% [44].

**Figure 2:**
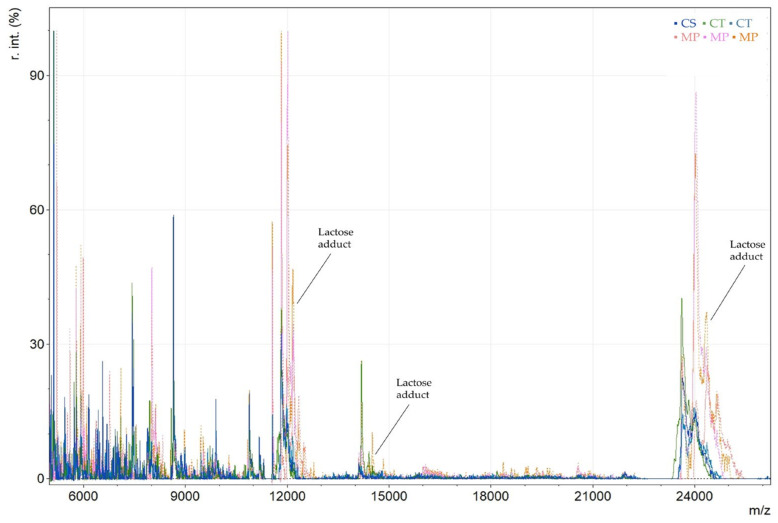
MALDI MS spectra of yogurt samples: cow milk yogurt (CT and CS, and cow milk powder (MP)).

Furthermore, spectra of cow milk powder (MP) were also compared with cow milk yogurt (CS and CT), revealing that caseins were the most abundant proteins. However, when comparing milk powder with yogurt, proteose peptone was not detected, and fewer proteolysis products were observed. Additionally, galactose or lactose adducts on proteins were identified in milk powder samples and may serve as characteristic markers of milk powder (Figure 2). Glycation is a heat-induced protein modification where sugars are attached to proteins during the processing and storage of UHT milk. Lactosylation is a process where lactose is attached to proteins and is mainly induced by thermal treatment [12]. The proteins with lactose adducts are expected to have a mass difference (Δm) of *m*/*z* 324, indicating one lactose molecule, or 648 and 972 for three and three lactose molecules, respectively.

As the main goal of the study was to authenticate yogurt samples, an extensive statistical analysis was performed, and our final conclusions were carried out through the aid of PCA-based models, such as PLS-DA. These chemometric models can efficiently assign specific markers to each yogurt type and to milk powder and detect the fraudulent addition of cow milk powder in yogurts.

### 3.1. Discrimination of Milk Animal Origin in Yogurt Samples (Cow/Goat/Ewe)

**Figure 3:**
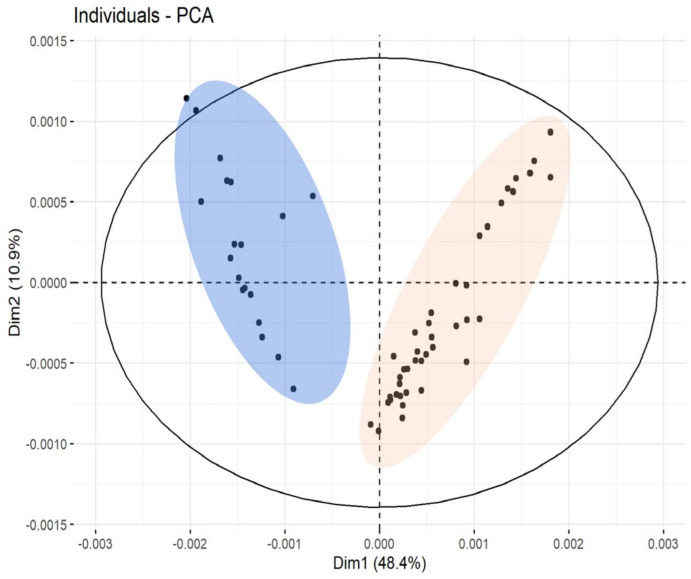
PCA score plot of cow, goat, and ewe milk yogurt samples.

The first dataset, containing a total of 60 spectra (from 49 distinct samples), was formed from the yogurt samples of different milk animal origins. The data were initially evaluated using an unsupervised PCA. As illustrated in Figure 3, the PCA model was built using two PCs that explain 59.3% of the total variation, meaning that a supervised method based on PCA models may be successful. However, with the applied method, this dataset was grouped into two clusters instead of the expected three: one for cow milk yogurt and one for goat and ewe milk yogurt. This result can be explained by the fact that goats and ewes are both ruminants belonging to the subfamily *Caprinae*, which gives them a similar protein profile. In contrast, cows belong to the subfamily *Bovinae*, resulting in a different protein composition. For this reason, some studies require the detection of species-specific markers at the peptide level to achieve a complete differentiation between goat and ewe milk [23,45].

**Figure 4:**
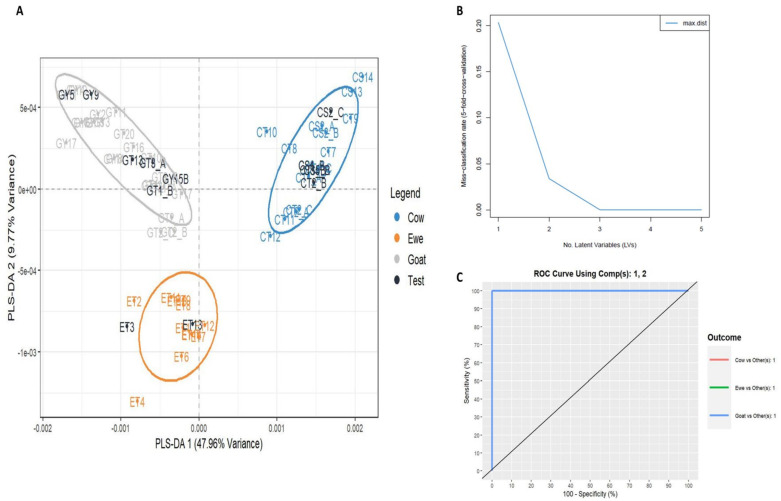
Plots derived from PLS-DA. (**A**) PLS-DA score plot for training and test samples, (**B**) latent variables, and (**C**) ROC curve using two PCs.

**Figure 5:**
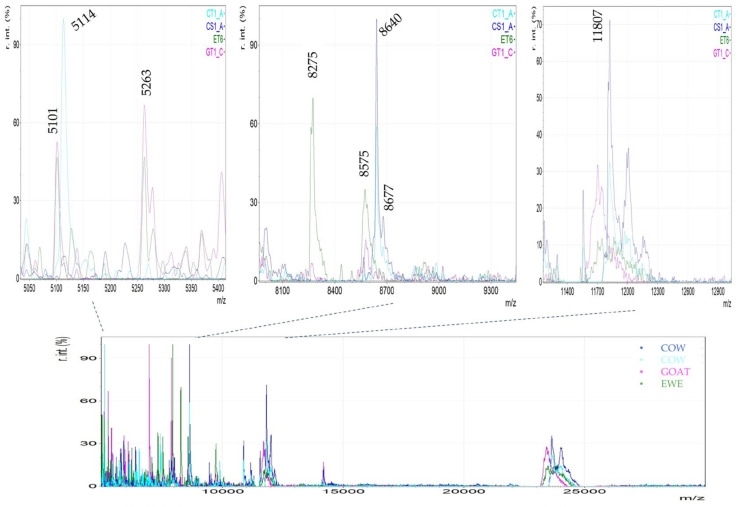
Typical MALDI mass spectra of yogurt samples. The magnified regions show the distinct protein patterns of each yogurt.

Next, a supervised analysis was performed for the prediction of unknown samples. To build the PLS-DA model, three groups of data were included in the training set: cow milk yogurt, ewe milk yogurt, and goat milk yogurt. Notably, “13” spectra (18%) were set as test samples to evaluate the model’s accuracy. The developed PLS-DA model showed a very low misclassification error rate (RMSEE = 0.068) and high discriminating power (Q^2^ = 0.947) (Figure 4A). Two latent variables were selected as the misclassification rate was lower than 0.05 (Figure 4B). The Receiver Operating Characteristic (ROC) curves, using the two PCs, show the trade-off between sensitivity (true positive rate) and “100—specificity” (false positive rate) and are illustrated in Figure 4C. The area under the curve (AUC) was calculated as 1.00 for all the pairs of the group tested, which means that 100% of the samples can be distinguished each time. From this analysis, the variables that contribute the most to the two selected PCs of the PLS-DA were identified as final important markers and were assigned to each type of yogurt based on animal milk origin. For instance, the variable with *m*/*z* 11,807 seemed to be characteristic of cow milk yogurt and was absent in ewe and goat milk yogurts. The variables with *m*/*z* 5101 and 5265 were presented in ewe and goat milk yogurts but not in yogurts from cow milk, while the variables with *m*/*z* 8275 and 8574 were only present in ewe milk yogurt. The MALDI spectra that depicted these observations are presented in Figure 5. In other studies, the main protein markers for milk animal origin discrimination were mostly the whey proteins (α-Lactalbumin and β-Lactoglobulin) among the other caseins and unknown proteins [8,33,46]. In our case, the most important discriminating variables seemed to be casein fractions. It is worth stressing the fact that the occurrence of *m*/*z* 8575 as a protein marker of ewe milk origin was also reported in the work of Sassi et al. [8] as an unknown ewe milk marker. The variable with *m*/*z* 11,807 was identified as the γ2-casein of cow milk [8,45,47], while the proteins of lower molecular weight (*m*/*z* 5101 and 5265) have not been reported before as markers of milk animal origin. All the important markers for yogurt animal origin speciation that were revealed from the statistical treatment are presented in Table 1.

### 3.2. Discrimination of Greek Cow Milk Yogurt Technological Process (Strained/Traditional)

**Figure 6:**
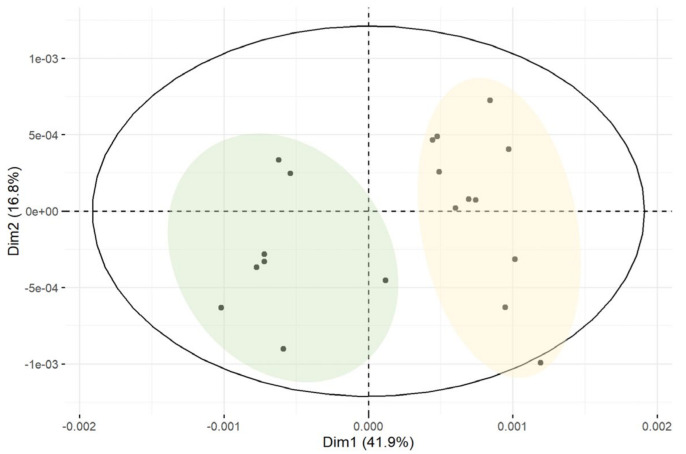
PCA score plot of cow milk traditional and cow milk strained yogurt samples.

During the initial visual observation of the samples of cow milk yogurt, differences were observed in their protein profile. For this reason, in the unsupervised PCA method, only the dataset of cow milk yogurt samples (21 unique spectra from 15 distinct samples) was used. In the PCA performed, two PCs explained 58.7% of the total variation in the dataset. The samples were successfully divided into two groups, representing the cow milk traditional yogurt (CT) and the cow milk strained yogurt (CS) (Figure 6); thus, the technological process of yogurt production differentiates the protein profile of yogurt.

**Figure 7:**
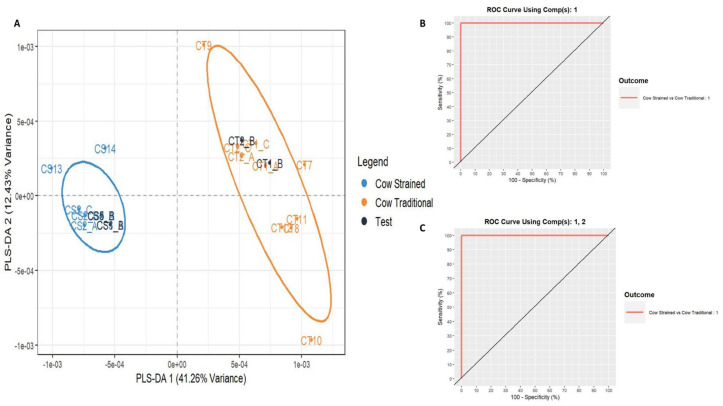
Plots derived from PLS-DA. (**A**) PLS-DA score plot for training and test samples, (**B**) ROC curve using one PC, and (**C**) ROC curve using two PCs.

Similarly to Section 3.1, a supervised PLS-DA analysis followed. Two groups of data were included in the training set of the PLS-DA model: CT and CS. Notably, 16% of the samples (four spectra) were set as test samples to evaluate the model’s accuracy. The developed PLS-DA model showed a very low misclassification error rate (RMSEE = 0.057) and high discriminating power (Q^2^ = 0.938) (Figure 7). The ROC curves, either using one or two PCs, are illustrated in Figure 7B,C. The AUC for the pair tested was calculated as 1.00 in both cases, meaning that 100% of the samples can be distinguished each time.

**Figure 8:**
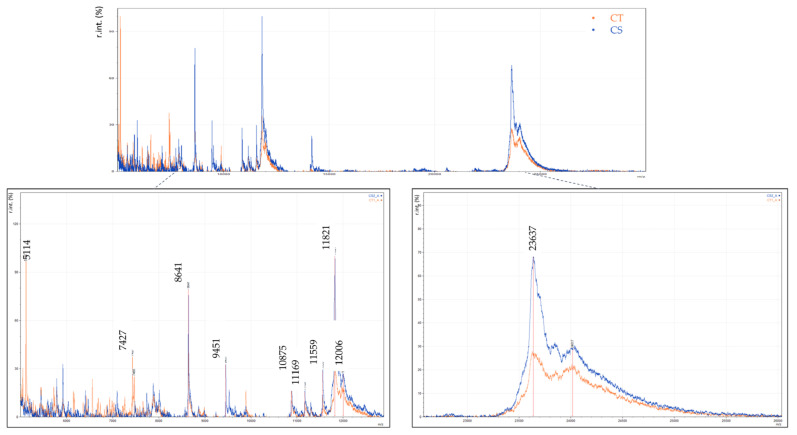
MALDI mass spectra of yogurt samples. The magnified regions show distinct protein patterns and characteristic variables of cow milk traditional yogurt (CT) and cow milk strained yogurt (CS).

The most significant variable as extracted from the PLS-DA has *m*/*z* 5115 and belonged only to the cow milk traditional yogurts (CT), as it was not detected on any strained yogurt (CS). This difference is depicted in the spectra presented in Figure 8. Other variables that contribute to the PCs are those with *m*/*z* 7427, 7466 and *m*/*z* 8639, 9451 which were present only in CTs and CSs, respectively. The variables with *m*/*z* 11,821, 11,856, 12,006, and 23,612 were more intense on strained yogurts than on traditional yogurts (Figure 8). The specific markers recognized for each yogurt type are also listed in Table 2.

### 3.3. Discrimination of Cow Milk Powder and Cow Milk Traditional Yogurt

In this part of the study, the protein profiles of cow milk yogurt and cow milk powder were thoroughly compared. Based on the existing literature, lactose adducts were expected on the milk proteins of powdered milk, due to extensive thermal treatment during its production. Firstly, the protein profile of milk powder from different manufacturers was examined to include fluctuations coming from different animal breeds and the technological process of milk powder production.

**Figure 9:**
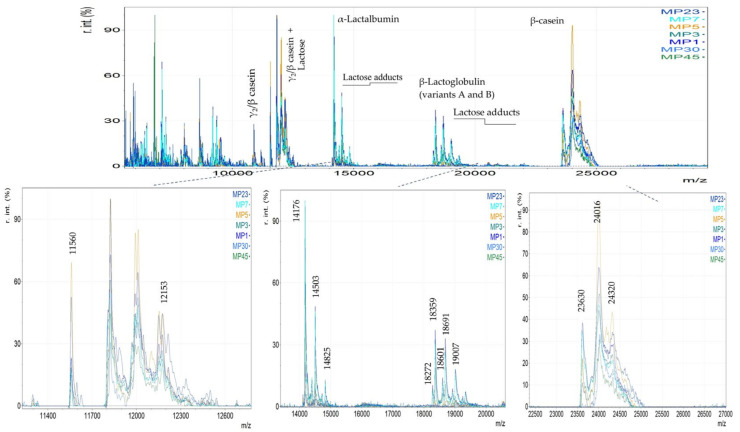
MALDI mass spectra of cow milk powder samples. The magnified regions show distinct protein patterns that were identified in the milk powder (MP).

In Figure 9, typical spectra of milk powders are illustrated. The milk powder spectra have peaks in the range of *m*/*z* 5000 to 9000, of lower abundance compared with yogurt spectra (Figure 2), indicating fewer proteolysis products [12]. This difference could be attributed to the fermentation process of yogurts. Moreover, whey proteins (α-Lactalbumin and β-Lactoglobulin) were detected in part of the samples. In this case, covalent adducts on both whey proteins were observed. Adducts were attributed to the lactosylation of proteins induced by milk’s thermal treatment causing Maillard reactions. In the mass range of α-Lactalbumin, four components were detected at *m*/*z* 14,176, 14,506, 14,825, and 15,143 (Figure 9). These peaks were identified as adducts of one, two, and three molecules of lactose on the intact protein, as the mass differences are 330, 648, and 967, respectively [8,32,33,48]. Similarly, in the mass range of β-Lactoglobulin mono-, di-, and tri-lactosylated forms were detected on the two variants of the intact protein with *m*/*z* 18,281, 18,600, 18,923, and 19,246 for variant A and *m*/*z* 18,358, 18,689, 19,009, and 19,343 for variant B.

**Figure 10:**
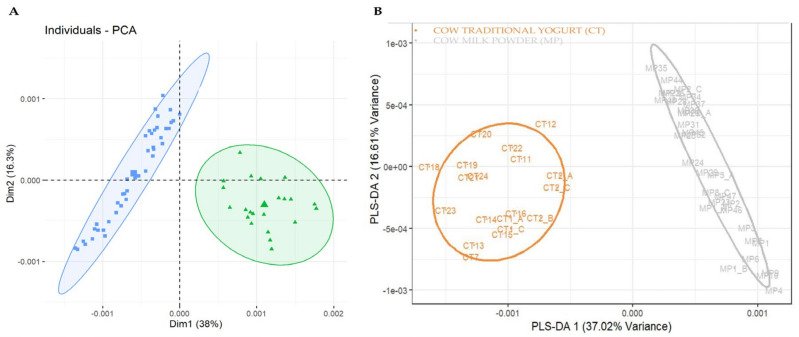
Plots of pairwise PCA (**A**) and PLS-DA (**B**) of cow traditional yogurt and cow milk powder.

For further analysis of the differences between spectra of milk powder (MP) and cow milk traditional yogurt (CT), similar statistical tools were used. A dataset of 64 spectra (from the 8 CT samples and the 19 MP samples) was used for unsupervised PCA. From this analysis, the samples were correctly grouped into the two expected groups with two PCs that were capable of explaining 54% of the total variance (Figure 10). From the PLS-DA that followed, (Figure 10) markers of the cow milk powder were revealed and are listed in Table 3. Specifically, according to the literature, the *m*/*z* value 11,990 has also been detected as an unknown protein of raw milk. The *m*/*z* value 8006 has been described as unknown and *m*/*z* 12,150 as γ_2_-casein with a lactose adduct and have both been assigned as markers of thermal treatment in UHT milk and powdered milk [8]. Finally, the *m*/*z* values 24,011 and 24,314 that were identified, for the first time, as indicators of thermal treatment could be attributed as glycation and/or lactosylation products of as1-casein.

### 3.4. Adulteration Study: Detection of Cow Milk Powder Addition in Cow and Goat Milk Traditional Yogurt to the Level of 1%

**Figure 11:**
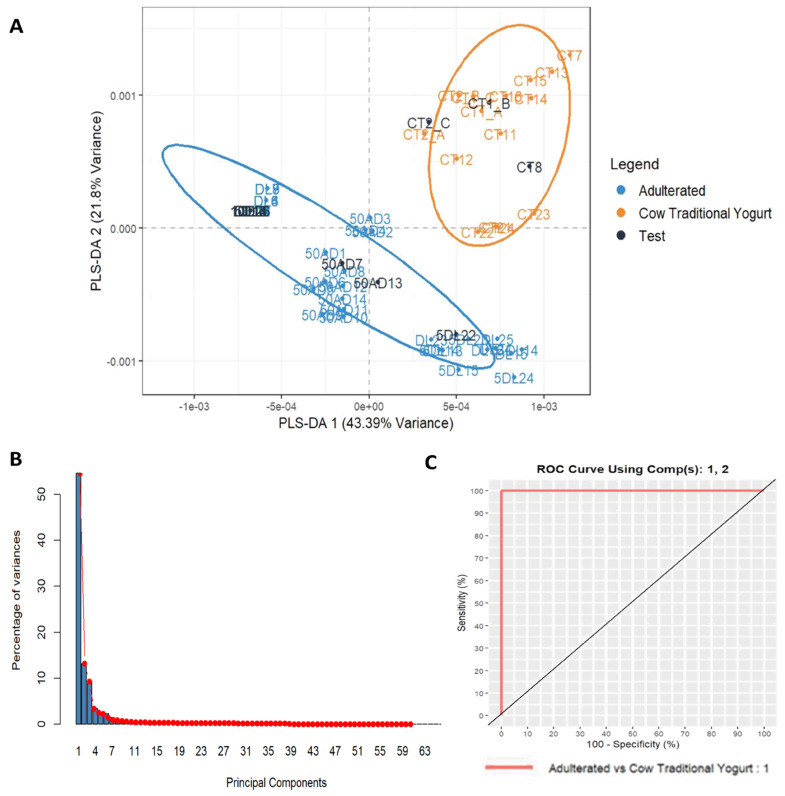
Plots derived from PLS-DA. (**A**) PLS-DA score plot for training and test samples for adulterated cow milk traditional yogurt with cow milk powder, (**B**) scree plot: principal components explained variances, and (**C**) ROC curve using two PCs.

**Figure 12:**
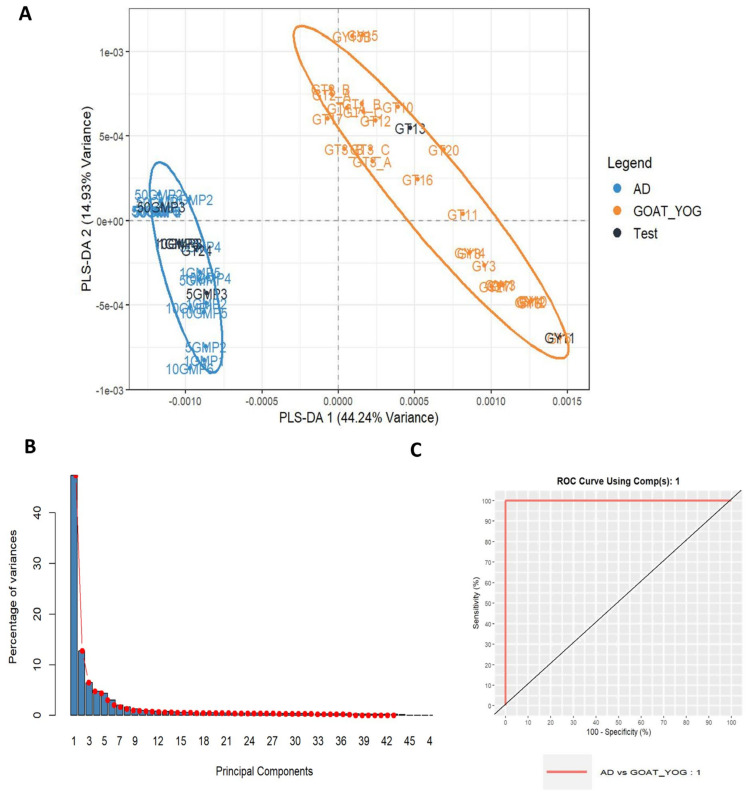
Plots derived from PLS-DA. (**A**) PLS-DA score plot for training and test samples for adulterated goat milk yogurt with cow milk powder, (**B**) scree plot: principal components explained variances, and (**C**) ROC curve using one PC.

The possibility of recognizing nondeclared cow milk powder in cow milk (CT) and goat milk (GT/GY) yogurts was tested by analyzing binary mixtures of 1, 5, 10, and 50% *w*/*w*. In total, 73 spectra (from 8 CT samples and 24 adulterated samples) were collected for the adulteration study of cow milk yogurt and 53 spectra (from 23 GT and 21 adulterated samples) for goat milk yogurt. Spectra were processed similarly as above and subjected to PCA for an initial descriptive approach. In both cases, two PCs explained more than 60% of the total variance, suggesting that the supervised model will successfully predict the group of unknown samples. Next, PLS-DA models were developed to predict cow milk powder addition in the two types of yogurts (Figure 11 and Figure 12). In particular, 13% of the spectra collected (10 spectra for the CT adulteration study and 7 spectra for the GT/GY adulteration study of all adulteration levels) were used as a test set. The developed PLS-DA models for cow milk yogurt and goat milk yogurt adulteration showed a low misclassification error rate (RMSEE = 0.119 and 0.063, respectively) and high discriminating power (Q^2^ = 0.865 and 0.968, respectively). Two latent variables were selected since the corresponding misclassification error rate was lower than 0.05 in a 5-fold cross-validation. The use of additional variables would not further improve classification accuracy. The calculated AUCs from the ROC curves was 1.00 for the pairwise adulterated CTs and cow milk traditional yogurts using two PCs and for the pairwise adulterated GT/GYs and goat milk yogurts using even one PC (Figure 11 and Figure 12). These results demonstrate that the model can efficiently predict the fraudulent addition of cow milk powder to cow milk and goat milk yogurts for all the investigated levels of adulteration.

**Figure 13:**
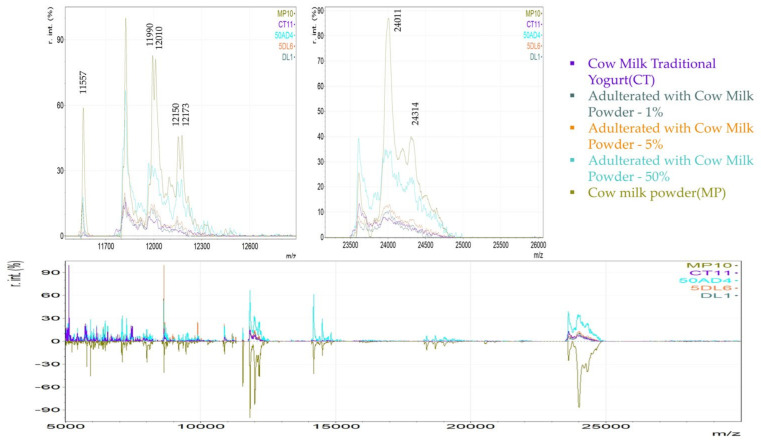
MALDI mass spectra derived from cow milk powder (MP), cow traditional milk yogurt (CT), and adulterated CT with MP in the levels of 1, 5, and 50%.

**Figure 14:**
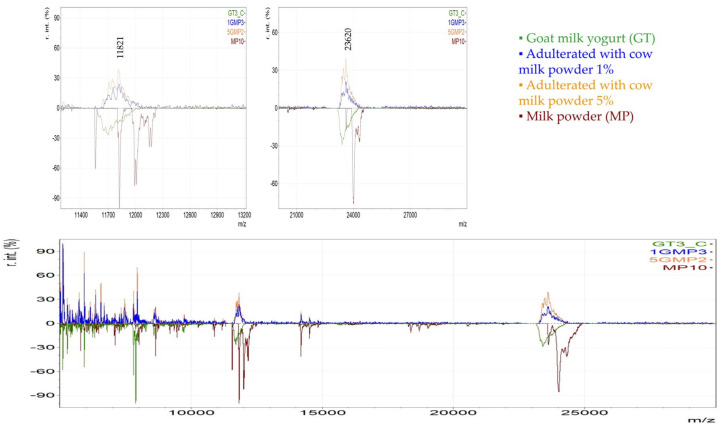
MALDI mass spectra derived from cow milk powder (MP), goat milk yogurt (GT), and adulterated GT with MP in the level of 1 and 5%.

For illustration purposes, in Figure 13 and Figure 14, an optical comparison between the spectra of authentic cow milk yogurt, goat milk yogurt, powdered milk, and adulterated respective samples is presented. The magnified regions highlight the variables identified as important markers to detect milk powder in yogurt. For instance, it is shown that the variables with *m*/*z* 11,990, 12,010, 12,150, and 12,173 were abundant in cow milk powder and in the adulterated CT samples even in the lowest level of 1%, while they are not found in cow milk traditional yogurt. Similarly, a lactose adduct in β-casein (*m*/*z* 24,314) was only detected in cow milk powder and in the adulterated samples of all levels and not in the cow milk traditional yogurt (Figure 13). In this case, both the yogurt and the milk powder originate from the same animal species. Therefore, the observed differences mainly reflect the effect of different thermal treatments. In the case of goat milk yogurt adulterated with cow milk powder, the proteomic profile is influenced by the introduction of cow-specific protein markers of the milk powder. Thus, two representative variables of cow milk powder with *m*/*z* 11,821 and 23,620 that were detected in the adulterated GT samples of all levels, including the 1%, and not in the goat milk yogurt are presented in Figure 14.

## 4. Conclusions

In this work, Greek yogurt’s intact protein profile was thoroughly investigated using MALDI-TOF MS. Using chemometric models, such as supervised PLS-DA, specific protein markers for Greek cow, goat, and ewe milk yogurt were identified. It should be highlighted that, for the first time, Greek cow milk yogurts were successfully classified as strained (set) and traditional, as characteristic differences in their protein profiles were recorded. Finally, the cow milk powder protein profile was also examined, and heat treatment patterns were identified in its protein profile. As part of an adulteration study of cow and goat milk yogurt with cow milk powder, the addition of cow milk powder to yogurt was detected to be as low as 1%. Finally, this is the first study of Greek yogurt’s protein profile and can be considered an important asset for Greek yogurt recognition as a PDO product.

## Figures and Tables

**Table 1:** Protein markers recognized for yogurt milk animal origin speciation.

Description	Experimental *m*/*z* Value	Literature
Cow milk yogurt		
Unknown	5115	
Unknown	5778	
Unknown	6402	
Unknown	7427	
Unknown	7466	
proteose peptone (fragment of β-Casein)	8640	
Unknown	10,879	
γ2-casein (fragment of β-Casein)	11,552	[8]
γ3-casein (fragment of β-Casein)	11,560	[8]
γ2-casein (fragment of β-Casein)	11,807	[8,48]
γ2-casein (fragment of β-Casein)	11,821	[8]
γ2-casein (fragment of β-Casein)	11,845	
as1-casein	23,608, 23,628, 23,647	[8]
Ewe milk yogurt		
Unknown	5101	
Unknown	5265	
Unknown	7940	
Unknown	8275	
Unknown	8574	[8]
Goat milk yogurt		
Unknown	5101	
Unknown	5265	
Unknown	5280	
Unknown	5406	
Unknown	7528	
Unknown	7795	
Unknown	7880	
Unknown	8589	
γ2-casein (fragment of β-Casein)	11,680	
γ2-casein (fragment of β-Casein)	11,700	
as1-casein (broad peak)	23,348, 23,392, 23,408	

**Table 2:** Protein markers recognized for cow milk yogurt technological processing speciation.

Description	Experimental *m*/*z* Value	Literature
Cow milk traditional (set) yogurt		
Unknown	5115	
Unknown	5124	
Unknown	5717	
Unknown	6143	
Unknown	6552	
Unknown	7427	
Unknown	7466	
γ2-casein (fragment of β-Casein)	11,807	[8,45,47]
Cow milk strained yogurt		
proteose peptone (fragment of β-Casein)	8639	
Unknown	9451	
Unknown	10,877	
γ2-casein (fragment of β-Casein)	11,807	

**Table 3:** Protein markers recognized for milk powder detection in cow and goat milk traditional yogurt from PLS-DA.

Description	Experimental *m*/*z* Value	Literature
Cow milk powder		
Unknown	8006	[8]
γ2-Casein (fragment of β-Casein)	11,557	[8]
Unknown	11,990	[8]
Unknown	12,010	
γ2casein + lactose	12,150	[8]
Unknown	12,173	
α-Lactalbumin	14,176	[8,33]
α-Lactalbumin + Lactose	14,506	[32,33,48]
α-Lactalbumin + 2 × Lactose	14,825	[32,33,48]
α-Lactalbumin + 3 × Lactose	15,143	[32,33,48]
β-Lactoglobulin (var 1, 2)	18,281, 18,358	[32,33,48]
β-Lactoglobulin (var 1, 2) + Lactose	18,600, 18,689	[32,33,48]
β-Lactoglobulin (var 1, 2) + 2 × Lactose	18,923, 19,009	[32,33,48]
β-Lactoglobulin (var 1, 2) + 3 × Lactose	19,246, 19,343	[32,33,48]
as1-casein	23,620	[33,48]
β-casein	24,011	
β-casein + Lactose	24,314	

## Data Availability

The data presented in this study are available upon request from the corresponding author due to the large and complex nature of the dataset, which makes public sharing impractical.
